# *cxcl12*-engineered endothelial progenitor cells enhance neurogenesis and angiogenesis after ischemic brain injury in mice

**DOI:** 10.1186/s13287-018-0865-6

**Published:** 2018-05-11

**Authors:** Yaning Li, Shuang Chang, Wanlu Li, Guanghui Tang, Yuanyuan Ma, Yanqun Liu, Fang Yuan, Zhijun Zhang, Guo-Yuan Yang, Yongting Wang

**Affiliations:** 1 School of Biomedical Engineering and Shanghai Jiao Tong University, Affiliated Sixth People’s Hospital, Shanghai Jiao Tong University, 1954 Hua Shan Road, Shanghai, 200030 China; 2 Department of Neurology, Ruijin Hospital, School of Medicine, Shanghai Jiao Tong University, Shanghai, 200030 China

## Abstract

**Background:**

Ischemic stroke causes a multitude of brain damage. Neurovascular injury and myelin sheath degradation are two manifestations of ischemic brain damage. Therapeutic strategies aiming only at repairing the neural components or the vessels cannot efficiently restore neurological function. Endothelial progenitor cells (EPCs) have the advantages of both promoting angiogenesis and secreting trophic factors that would promote neurogenesis. Chemokine *cxcl12* gene therapy has also been shown to promote angiogenesis, neurogenesis, and remyelination, attracting EPCs, neural progenitor cells, and oligodendrocyte progenitor cells (OPCs) to the injured sites of the brain. In this work, we tested whether these two therapeutics can be combined by genetically engineering the EPCs with *cxcl12* to harness the synergistic effects of these two interventions.

**Methods:**

We used lentivirus (LV) to deliver *cxcl12* gene into human umbilical cord blood EPCs to generate the engineered CXCL12-EPCs, which were then delivered into the perifocal region at 1 week after permanent middle cerebral artery occlusion to investigate the effects of CXCL12-EPCs on the functional recovery and angiogenesis, neurogenesis, and remyelination in ischemic stroke mice. *Green fluorescent protein* (*gfp*) gene-modified EPCs and LV-CXCL12 gene therapy were used as controls.

**Results:**

CXCL12-EPC treatment significantly reduced brain atrophy and improved neurobehavioral function at 5 weeks after brain ischemia. The treatment resulted in increased blood vessel density and myelin sheath integrity, and promoted neurogenesis, angiogenesis, and the proliferation and migration of OPCs. In-vitro data showed that CXCL12-EPCs performed better in proliferation and tube formation assays and expressed a higher level of vascular endothelial growth factor compared to GFP-EPCs.

**Conclusions:**

The synergistic treatment of CXCL12-EPCs outperformed the single therapies of GFP-EPCs or LV-CXCL12 gene therapy in various aspects related to post-ischemic brain repair. *cxcl12*-engineered EPCs hold great potential in the treatment of ischemic stroke.

## Background

Cerebral ischemia causes a cascade of detrimental events, including excitotoxicity, peri-infarct polarization, blood–brain barrier impairment, inflammation, and apoptosis, resulting in a multitude of injuries among which neurovascular damage and myelin sheath degradation are two signature events [1–4], which together result in failed or false signal transduction at the cellular level, consequently causing the various deficits of neural behavioral function in patients. Angiogenesis, neurogenesis, remyelination, and neuroplasticity are very critical components of functional recovery. Efficient recovery of neurological functions requires consideration of both the neural and vascular components. The stroke pathology is a long process, with increased cell death as early as 30 min, and persists for 4 weeks after ischemic injury as evidenced by DNA fragmentation [5]. MRI parameters also showed that the new lesion area, which presents the damaged brain area emerging 1 day after ischemia, continues to increase until about 6 months post ischemia [6]. Compared to the relatively narrow treatment window of thrombolytic tissue plasminogen activator (tPA) [7] and endovascular thrombectomy, the longer subacute phase presents additional opportunities for intervention.

Endothelial progenitor cells (EPCs) were first isolated in 1997 and found to incorporate into sites with active angiogenesis [8]. The increase of blood circulating EPCs after acute ischemic stroke is associated with preferred functional outcomes and reduced infarction [9]. The lowered number of EPCs is an independent risk factor for poor outcome in patients with acute ischemic stroke [10, 11]. These findings suggest that EPCs may participate in brain repair after ischemic stroke. Previous studies have shown that systemic delivery of EPCs protects the brain against ischemic injury, promotes neurovascular repair, and improves long-term neurobehavioral outcomes [12–14]. During EPC-mediated neuroprotection, chemokine CXCL12 has been shown to play critical roles [15].

Chemokine CXCL12, also known as stromal derived factor-1, was first found to attract early-stage B-cell precursors by binding with its receptor CXCR4 [16]. CXCL12 mediates vascular development by homing circulating EPCs to the site of active angiogenesis and vasculogenesis [17–19]. CXCL12 is a key regulator in the development of the nervous system, mainly by attracting the migration of CXCR4-expressing neural progenitor cells (NPCs) and guiding axon growth [20–22]. During myelination and remyelination processes in the embryonic and postnatal central nervous system, CXCL12 also plays important roles in regulating the survival and outward migration of oligodendrocyte progenitor cells (OPCs) [23–26]. In vitro, CXCL12 has been shown to attenuate the apoptosis of EPCs in serum starvation conditions [27, 28], and prevent EPC senescence and enhance reendothelialization in injured arteries [29]. These data suggest that EPCs and CXCL12 both hold the potential to treat brain injury.

Previous work demonstrated that both EPC therapy and *cxcl12* gene therapy yielded positive results in ischemic stroke mice models [14, 30]. In addition, we also observed that *cxcl12* modification of EPCs improved their proliferation and tube formation [31]. Gene engineering of stem cells has been shown to augment their regenerative abilities. The recent clinical trials using modified bone marrow-derived mesenchymal stem cells in the recovery phase also yielded promising outcomes [32]. We hypothesized that *cxcl12* engineering of EPCs will afford synergistic effect in improving stroke outcomes.

In this work, we investigated the treatment efficacy of CXCL12-EPCs in the permanent middle cerebral artery occlusion model (pMCAO) of mice. We used lentivirus to engineer EPCs with the *cxcl12* gene or *gfp* gene. We then delivered CXCL12-EPCs, GFP-EPCs (as stem cell therapy control), LV-CXCL12 (as gene therapy control), and PBS (as vehicle control) into the perifocal area through stereotactic injection at 1 week after pMCAO. The effects of CXCL12-EPC treatment on the functional recovery, angiogenesis, neurogenesis, and remyelination were investigated.

## Methods

### Experimental protocol

A total of 58 adult male Institute of Cancer Research (ICR) mice underwent pMCAO surgery. Animals were trained on rotarod for 3 consecutive days before MCAO surgery. Thirty-nine of them survived MCAO beyond 1 week. Three animals were excluded due to the lack of obvious neurological deficit. Thirty-six animals with similar modified neurological severity score (mNSS) at 7 days after surgery were randomly assigned into four different treatment groups, with 11 mice in the PBS group, nine mice in the LV-CXCL12 group, eight mice in the GFP-EPC group, and eight mice in the CXCL12-EPC group. At 7 days after pMCAO surgery, the ischemic mice received either PBS, LV-CXCL12, GFP-EPCs, or CXCL12-EPCs via stereotactical injection into the perifocal region. The mNSS and rotarod performance were followed for 5 weeks after pMCAO. BrdU (Sigma, St. Louis, MO, USA) dissolved in normal saline at a concentration of 10 mg/ml was injected intraperitoneally at a dose of 50 mg/kg each day from 28 to 35 days after pMCAO for 7 consecutive days before the animals were sacrificed at 5 weeks.

### pLV-CXCL12-IRES-GFP vector construction and LV-CXCL12 production

**Fig. 1:**
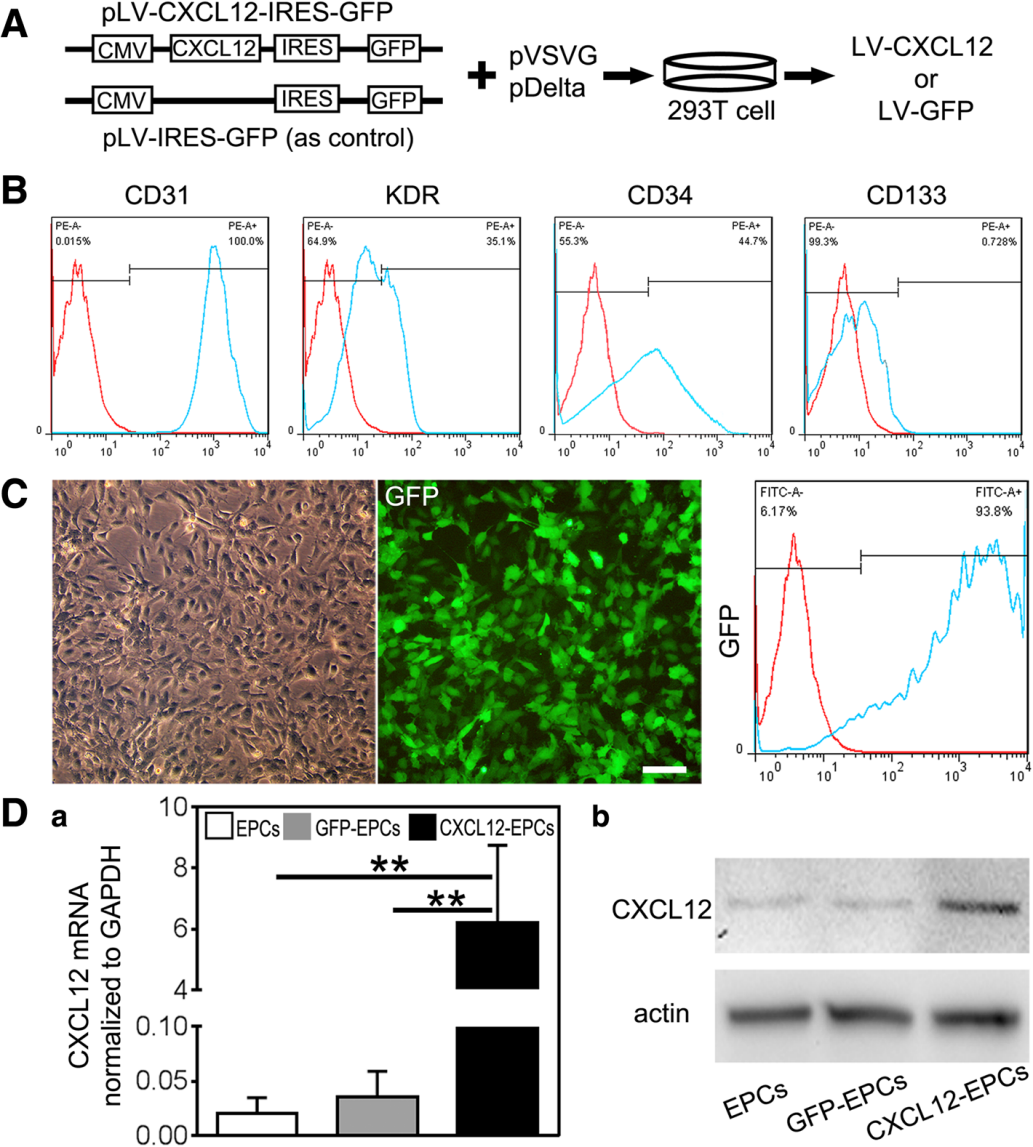
LV-CXCL12 virus successfully transfected EPCs in vitro. **A** Clone of pLV-CXCL12-IRES-GFP plasmid by inserting mouse *cxcl12* cDNA sequence in pLV-IRES-GFP plasmid, then cotransfected 293 T cells with pVSVG and pDelta plasmid to package LV-CXCL12 and LV-GFP virus. **B** Flow cytometry to characterize human umbilical cord blood-derived EPCs by cell surface markers CD31, KDR, CD34, and CD133. **C** LV-CXCL12-transfected EPCs in bright field (left), fluorescent field (middle), and flow cytometry (right) to identify transfection efficacy of CXCL12-EPCs. Scale bar: 100 μm. **D** Real-time PCR and western blot analysis to detect CXCL12 mRNA (**a**) and protein (**b**) expression in EPCs, GFP-EPCs, and CXCL12-EPCs. *n* = 3, technical replica. Data presented as mean ± SD. **p < 0.01. GFP green fluorescent protein, EPC endothelial progenitor cells, GFP-EPC endothelial progenitor cell modified by *gfp* gene, CXCL12-EPC endothelial progenitor cell modified by *cxcl12* gene

pLV-CXCL12-IRES-GFP vector was subcloned by inserting mouse *cxcl12* cDNA into the multiple-cloning site of pLV-IRES-GFP plasmid. pLV-CXCL12-GFP was cotransfected with pVSVG and pDelta plasmids into 293 T cells by calcium phosphate precipitation (Fig. 1A). The viruses were further purified by density gradient ultracentrifugation in 20% sucrose in PBS. LV-GFP was simultaneously prepared as control following a published protocol [33].

### EPC isolation and identification

EPCs were isolated from human umbilical cord blood obtained from the International Peace Maternity and Child Health Hospital, Shanghai, China. This procedure was approved by the Ethics Committee of Shanghai Jiao Tong University, Shanghai, China. EPC isolation and identification were carried out as described previously [13, 31] and characterized similar to that reported by other groups [34]. Briefly, monocytes were isolated from umbilical cord blood by centrifugation with lymphocyte separation medium (MP Biomedicals, Santa Ana, CA, USA) and washed twice with M199 medium (Hyclone, Logan, UT, USA). The cells were resuspended in EGM-2 Bullet kit medium (Lonza, Anaheim, CA, USA), seeded in a six-well plate (1 × 10^7^ cells per well) coated with human fibronectin (Sigma), and incubated at 37 °C with 5% CO_2_. After growing to about 80% confluence, the cells were trypsinized and passaged.

The EPCs were identified by flow cytometry (FACScalibur; BD Biosciences, Franklin Lakes, NJ, USA). For this purpose, EPCs were incubated with fluorescent antibodies of CD31-PE, CD34-PE (eBioscience, San Diego, CA, USA), KDR-PE (BD Biosciences), or CD133-PE (Miltenyi Biotec, Shanghai, China) in the dark for 30 min, and washed twice by PBS before being loaded onto the FACS for analysis.

EPCs at 80% confluence were transfected with LV-CXCL12 (CXCL12-EPCs) or LV-GFP (GFP-EPCs) at the third passage. The transfection efficiency was identified by flow cytometry for GFP-positive cells. All experiments were performed using the fifth passage of EPCs.

### EPC proliferation and tube formation assay

EPC proliferation was examined using the cell counting kit-8 (CCK-8; Dojindo, Kumamoto, Japan) [35]. EPCs (1 × 10^4^ cells), GFP-EPCs, and CXCL12-EPCs were incubated with the mixture of CCK-8 and EGM-2 medium (1:10) for 5 h in a 96-well plate. The media absorbance at 450 nm was then recorded using a micro-plate reader (BioTek, Chicago, IL, USA). Blank control was set by the absorption of the medium only.

The EPC tube formation assay was performed as described previously [13]. Matrigel (50 μl; BD Biosciences) was added to a 96-well plate and allowed to solidify at 37 °C for 30 min. Then 1 × 10^4^ EPCs, GFP-EPCs and CXCL12-EPCs were suspended in 100 μl EGM-2 medium and seeded in the matrigel-coated plate. After incubating for 6 h, the plate was examined with a microscope (Leica, Solms, Germany). The tube number in each well was counted by an investigator blind to the sample designation.

### CXCL12 and VEGF real-time polymerase chain reaction quantification

Total RNA from the EPCs, GFP-EPCs, and CXCL12-EPCs were isolated using TRIzol reagent (Invitrogen, Carlsbad, CA, USA) and suspended in 40 μl of RNase-free water according to the manufacturer’s protocol. The amplification was performed by a fast-real-time polymerase chain reaction (PCR) system (7900HT; ABI, Foster, CA, USA) using a SYBR Premix Ex Taq Kit (Takara, Dalian, China). CXCL12 and VEGF mRNA levels were normalized to the endogenous control GAPDH expression in triplicate. The primers used were as follows: mouse CXCL12, forward TGCATCAGTGACGGTAAACCA and reverse CACAGTTTGGAGTGTTGAGGAT; human VEGF, forward GAGGAGCAGTTACGGTCTGTG and reverse TCCTTTCCTTAGCTGACACTTGT; and human GAPDH, forward CTGGGCTACACTGAGCACC and reverse AAGTGGTCGTTGAGGGCAATG.

### CXCL12 and VEGF western blot assay

Proteins were extracted separately from EPCs, GFP-EPCs, and CXCL12-EPCs with RIPA (Millipore, Temecula, CA, USA). The western blot protocol was carried out as described previously [36]. The primary antibodies were CXCL12 and VEGF (1:1000; Abcam, Cambridge, MA, USA), and β-actin (1:1000; Santa Cruz Biotechnology, Santa Cruz, CA, USA).

### Permanent middle cerebral artery occlusion in mice

Adult male ICR mice (Sippr-bk, Shanghai, China) weighing 30 ± 2 g were anesthetized by ketamine/xylazine (100/10 mg/kg; Sigma-Aldrich, St Louis, MO, USA). MCAO was carried out as described previously [30]. Briefly, after isolation of the common carotid artery and external and internal carotid arteries, the left MCA was occluded by inserting a 6-0 nylon suture coated with silica gel. Body temperature was maintained at 37 °C throughout the surgery using a thermal blanket. Successful occlusion was verified by laser Doppler flowmetry (Moor Instruments, Axminster, UK).

### Neurobehavioral tests

An investigator blind to the experimental design carried out a rotarod test and neurological evaluations using the mNSS. Baseline values were generated by selecting a maximum of six trials before surgery. Rotarod test data were analyzed using the same method as that of the baseline values. Mice were examined up to 5 weeks after pMCAO. The rotarod test required mice to balance on a rotating rod. Mice were allowed 1 min for adaption on the rod, after which the rod was accelerated to 40 rpm over 2 min and the time spent on the rod was recorded. The mNSS of the animals was graded on a scale of 0–18, which is a composite of motor, reflex, and balance tests [37].

### LV-CXCL12 viral vector injection and GFP/CXCL12-EPC transplantation

One week after pMCAO, the mice were anesthetized and immobilized on a stereotaxic frame (RWD Life Science, Shenzhen, China). A total volume of 10 μl of PBS containing 5 × 10^7^ LV-CXCL12 viral particles or 3 × 10^5^ GFP/CXCL12-EPCs were injected stereotactically at a rate of 500 nl/min at 2 mm lateral to the bregma and 3 mm under the dura. The needle was maintained for 10 min before withdrawal. The bone hole was sealed with bone wax and the wound was stitched. After awakening from anesthesia, the mice were returned to their cages for long-term recovery.

### Brain atrophy measurement

Brains were removed and frozen immediately after sacrificing the mice. A series of 20-μm coronal sections 1.3 mm to −2.7 mm from the bregma were cut and mounted on glass slides. The sections were stained using a cresyl violet solution. The atrophic area was calculated by subtracting the cresyl violet stained area in the ipsilateral hemisphere from the whole area of the contralateral hemisphere using ImageJ software (NIH, USA). The atrophy volume was calculated using the formula $\sum \limits_1^n\left[\left({S}_n+\sqrt{S_n\ast {S}_{n+1}}+{S}_{n+1}\right)\ast \frac{h}{3}\right]$, where *S* represents the atrophic area (mm^2^) in each brain section and *h* represents the distance between adjacent brain slices that were stained, here *h* = 0. 2 mm.

### Immunohistochemistry

Brains were post-fixed for 4–5 h followed by 24 h of immersion in 30% sucrose in PBS, immediately frozen, and then sectioned using a cryostat (Leica, Solms, Germany). Then 20-μm coronal sections were cut. Floating coronal sections were collected in antigen protective solution, which contains 50% PBS, 30% glycerol, and 20% glycol.

Immunohistochemistry was performed according to the protocol described previously [30]. Care was taken to sample sections with similar anatomical features. The primary antibodies were: CD31 (1:200 dilution; R&D Systems, Minneapolis, MN, USA); NeuN (1:100 dilution) and platelet-derived growth factor α (PDGFRα; 1:200 dilution) (Millipore, Billerica, MA, USA); BrdU and DCX (1:200 dilution; Santa Cruz Biotechnology); and myelin basic protein (MBP; 1:300 dilution) (Abcam).

For BrdU staining, sections were first treated with 2 mol/L HCl for 30 min at 37 °C and then neutralized twice with 0.1 mol/L sodium borate (pH 8.5) for 10 min each. Sections were then treated with 0.3% Triton-100 in PBS for 10 min, blocked by 5% normal donkey serum, and incubated with anti-BrdU and anti-DCX (or anti-NeuN or anti-CD31) antibody at 4 °C overnight. Finally, the sections were incubated with proper secondary antibodies for 60 min at room temperature. Stained sections were mounted after rinsing. For biotinylated immunostaining, the brain sections incubated with the same primary antibodies were developed for the same amount of time.

### Cell and vessel counting

Four fields were selected randomly from the perifocal region at × 20 or × 40 objective. DCX^+^/BrdU^+^ and PDGFRα cells in the subventricular zone (SVZ) were counted for each image (DM2500; Leica Microsystems, Wetzlar, Germany). Sections incubated with the same primary antibodies were imaged under the same conditions by an investigator blind to sample designation. The quantity of BrdU^+^, DCX^+^/BrdU^+^, NeuN^+^/BrdU^+^, PDGFRα^+^ cells, CD31^+^ and CD31^+^/BrdU^+^ microvessels in the ipsilateral hemisphere were counted and quantified by an investigator blind to the experimental groups in the same manner. Four serial sections, spaced 400 μm apart (1.10 mm to −0.1 mm from the bregma), were selected from each animal.

### Integral optical density of MBP^+^ myelin sheath fluorescence

MBP staining intensity was computed as the mean integrated optical density (IOD) as described previously [38]. Briefly, four fields were randomly selected from the perifocal region at × 20 objective (DM2500; Leica Microsystems) under the same conditions. The images were automatically analyzed by the “Pathology” function of Image Pro Plus 6.0 (Media Cybernetics, Bethesda, MD, USA) for quantitative analysis, where IOD was calculated for arbitrary areas. Brain sections not incubated in the primary antibody were used to estimate the background staining. Four serial sections, spaced 400 μm apart, were selected from each animal and there were four animals in each group. The results were normalized to the IOD of PBS group.

### Statistical analysis

Parametric data analysis for the mNSS and the rotarod test was performed by repeated-measured two-way ANOVA followed by Tukey’s test. For nonrepeated data analysis, one-way ANOVA followed by Tukey’s test was used for three groups or more, and Student’s *t* test was performed for two groups using GraphPad Prism version 6.02 (GraphPad Software, Inc., La Jolla, CA, USA). All data were presented as mean ± SD. A probability value of *p* < 0.05 was considered statistically significant.

## Results

### LV-CXCL12 virus successfully transfected EPCs in vitro

To modify EPCs with the *cxcl12* gene in vitro, we constructed pLV-CXCL12-IRES-GFP plasmid by inserting *cxcl12* cDNA into pLV-IRES-GFP plasmid. pLV-CXCL12-IRES-GFP or pLV-IRES-GFP plasmid was used to cotransfect 293 T cells with pVSVG and pDelta plasmids to produce LV-CXCL12 and LV-GFP (as control) (Fig. 1A). Flow cytometry analysis showed that the isolated cells were approximately 100% CD31-positive, 35.1% KDR-positive, 44.7% CD34-positive, and 0.728% CD133-positive (Fig. 1B), which demonstrated that these isolated cells were EPCs. LV-CXCL12 virus efficiently transfected EPCs, with 93.8% cells being GFP-positive (Fig. 1C). Further, real-time PCR and western blot analysis showed that the expression of CXCL12 both at mRNA and protein levels were significantly increased in CXCL12-EPCs compared to EPCs and GFP-EPCs (Fig. 1D). The gene-modified CXCL12-EPCs, GFP-EPCs (as stem cell control), and LV-CXCL12 virus (as gene therapy control) were used in the following experiments.

### CXCL12-EPC transplantation improved neurobehavioral outcomes and reduced brain atrophy in ischemic mice

**Fig. 2:**
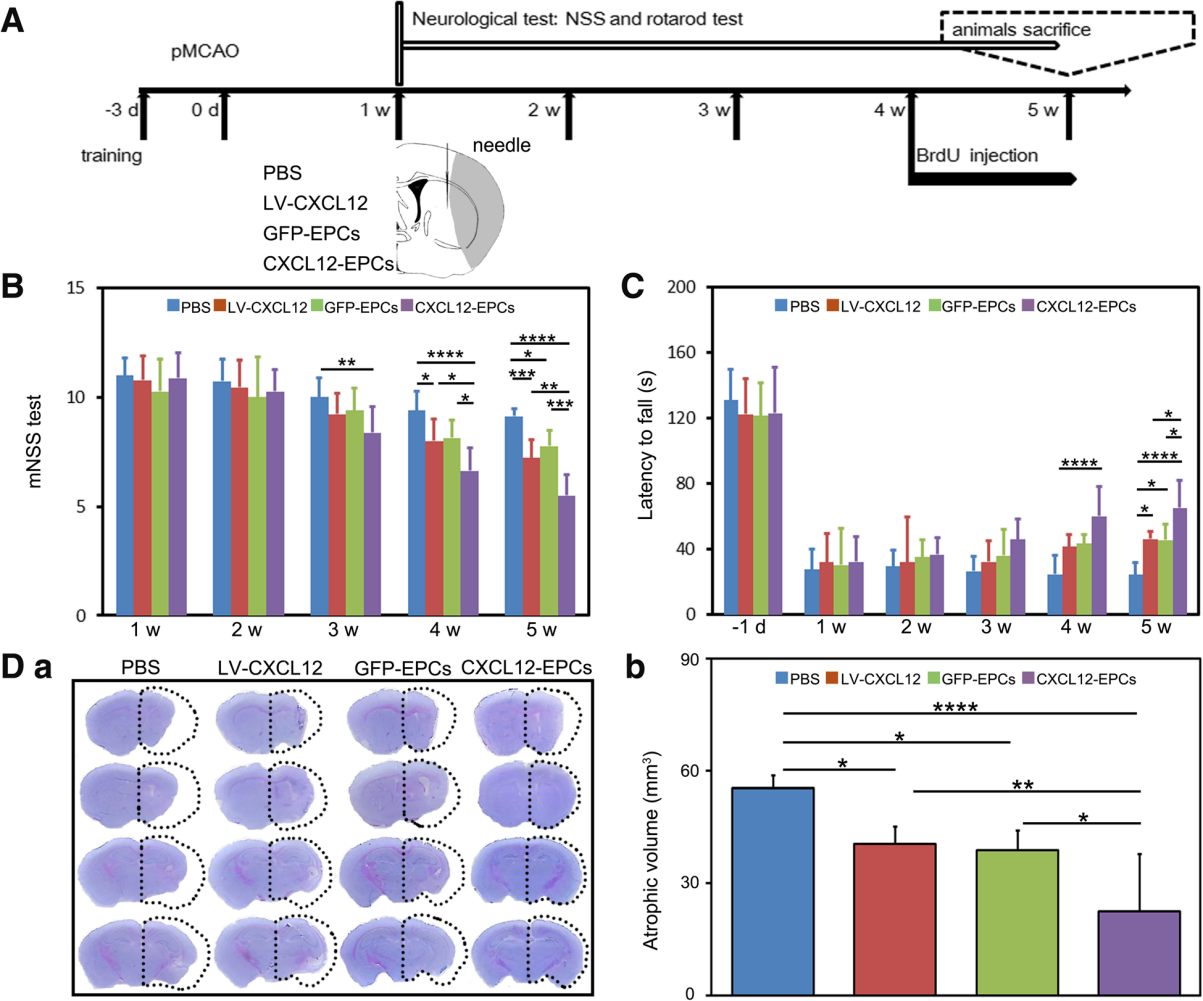
CXCL12-EPC transplantation improved neurobehavioral outcomes and reduced brain atrophy at 5 weeks in ischemic mice. **A** Experimental design. **B** mNSS evaluation in PBS, LV-CXCL12, GFP-EPC, and CXCL12-EPC groups (*n* = 8–11 mice/group). **C** Rotarod test in PBS, LV-CXCL12, GFP-EPC, and CXCL12-EPC groups (*n* = 8–11 mice/group). **D** (**a**) Representative micrographs of coronal sections stained by cresyl violet for brain atrophy at 5 weeks after ischemic stroke. (**b**) Quantification of brain atrophy (*n* = 6 mice/group) at 5 weeks after ischemic stroke. Data presented as mean ± SD. *p < 0.05, **p < 0.01, ***p < 0.001, ****p < 0.0001. pMCAO permanent middle cerebral artery occlusion, mNSS modified neurological severity score, d day, w week, PBS phosphate-buffered saline, GFP-EPC endothelial progenitor cell modified by *gfp* gene, GFP green fluorescent protein, CXCL12-EPC endothelial progenitor cell modified by *cxcl12* gene

The experimental plan is illustrated in Fig. 2A. To evaluate the roles of CXCL12-EPC transplantation in neurological outcomes, neurological assessments including the mNSS and the rotarod test were carried out for up to 5 weeks after pMCAO. The mNSS was significantly improved in the gene therapy and stem cell therapy groups at 5 weeks after ischemia compared to the PBS group (9.13 ± 0.35), with a better outcome in the CXCL12-EPC group (5.50 ± 0.93) compared to the LV-CXCL12 gene therapy group (7.22 ± 0.83) and the GFP-EPC cell therapy group (7.75 ± 0.71) (Fig. 2B). In parallel, the rotarod test showed that the motor function greatly improved in all therapeutic groups at 5 weeks after ischemia compared to the PBS group (24.63 ± 7.54 s), with a better improvement in the CXCL12-EPC group (65.25 ± 16.82 s) compared to the LV-CXCL12 group (46.22 ± 4.99 s) and the GFP-EPC group (45.75 ± 9.60 s) (Fig. 2C). Furthermore, we characterized whether CXCL12-EPC transplantation protected the brain from injury and found that the brain atrophy volume was reduced in the LV-CXCL12 (81.23 ± 9.16 mm^3^), GFP-EPC (77.54 ± 10.91 mm^3^), and CXCL12-EPC (44.83 ± 31.17 mm^3^) therapy groups compared to the PBS group (110.93 ± 6.79 mm^3^), with a smaller atrophic volume in the CXCL12-EPC group compared to the GFP-EPC and LV-CXCL12 treatment groups (Fig. 2D).

### CXCL12-EPC transplantation increased blood vessel density and promoted angiogenesis in ischemic mouse brain

**Fig. 3:**
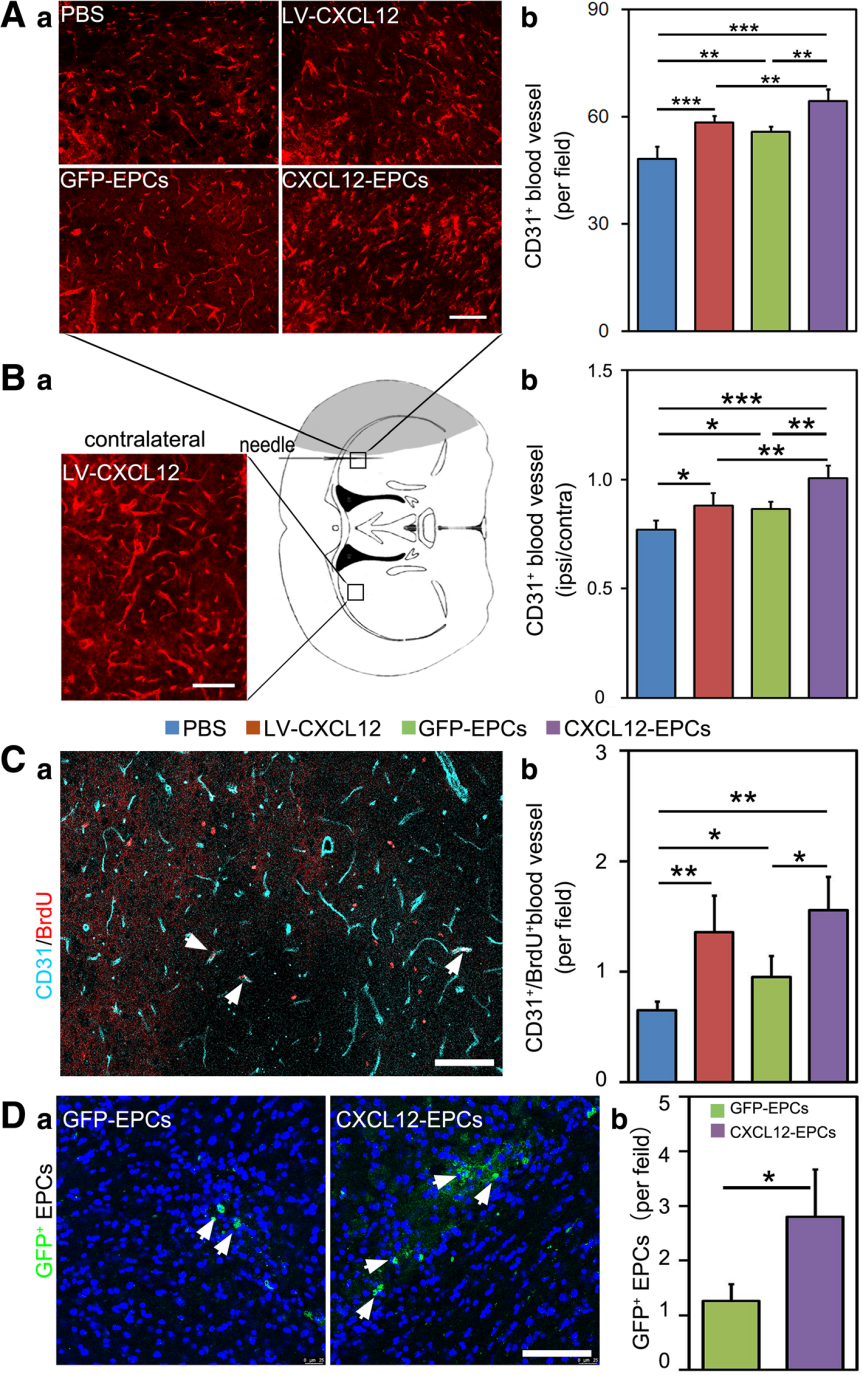
CXCL12-EPC transplantation increased blood vessel density and promoted angiogenesis in ischemic mouse brain. **A** (**a**) Photomicrographs of CD31^+^ microvessels in ipsilateral hemisphere in PBS, LV-CXCL12, GFP-EPC, and CXCL12-EPC groups at 5 weeks after MCAO. (**b**) Quantification of CD31^+^ microvessels in perifocal region 5 weeks after MCAO. **B** (**a**) Representative photomicrographs of CD31^+^ microvessels in contralateral hemisphere. Hollow box in brain diagram shows area of interest at 5 weeks after MCAO. (**b**) Ratio of ipsilateral/contralateral CD31^+^ microvessels at 5 weeks after MCAO. **C** (**a**) Representative photomicrographs of CD31 and BrdU immunostaining of ipsilateral hemisphere at 5 weeks after MCAO. Arrows indicate CD31^+^/BrdU^+^ colocalized newly formed microvessels. (**b**) Quantification of CD31^+^/BrdU^+^ newly formed microvessels in ipsilateral hemisphere at 5 weeks after MCAO. **D** (**a**) Representative image of GFP^+^ cells (white arrows) from GFP-EPC and CXCL12-EPC therapy mouse brain 4 weeks after transplantation. (**b**) Quantification of GFP^+^ EPCs in ipsilateral hemisphere in GFP-EPC and CXCL12-EPC-treated group 4 weeks after transplantation. Scale bar: 100 μm. *n* = 3–4 mice/group. Data presented as mean ± SD. *p < 0.05, **p < 0.01, ***p < 0.001. All markers presented by pseudo colors. PBS phosphate-buffered saline, GFP-EPC endothelial progenitor cell modified by *gfp* gene, GFP green fluorescent protein, CXCL12-EPC endothelial progenitor cell modified by *cxcl12* gene

CD31 immunostaining was performed to examine the blood vessel density after treatment. The density of CD31^+^ blood vessels in the perifocal area was significantly higher in all therapeutic groups compared to the PBS group, with more CD31^+^ blood vessels in the CXCL12-EPC cell therapy group compared to the LV-CXCL12 and GFP-EPC groups (Fig. 3A). The ratios of blood vessel in ipsilateral to contralateral hemisphere were also higher in all therapeutic groups compared to the PBS group, with the highest CD31^+^ blood vessel ratio in the CXCL12-EPC cell therapy group (Fig. 3B). We further examined whether CXCL12-EPC promoted angiogenesis by performing CD31 and BrdU double immunostaining. Data showed that the number of CD31^+^/BrdU^+^ blood vessels was increased in all therapeutic groups compared to the PBS group, with more CD31^+^/BrdU^+^ blood vessels in the CXCL12-EPC group compared to the GFP-EPC group (Fig. 3C), which indicated that the transplantation of CXCL12-EPCs not only increased blood vessel intensity, but also promoted angiogenesis in ischemic mouse brain. The transplanted CXCL12-EPCs exhibited a better cell viability in ischemic mouse brain than GFP-EPCs, which were supported by higher numbers of GFP-positive cells in the ischemic mouse brain in the CXCL12-EPC group at 4 weeks after transplantation (Fig. 3D).

### CXCL12-EPC transplantation promoted neurogenesis in ischemic mouse brain

**Fig. 4:**
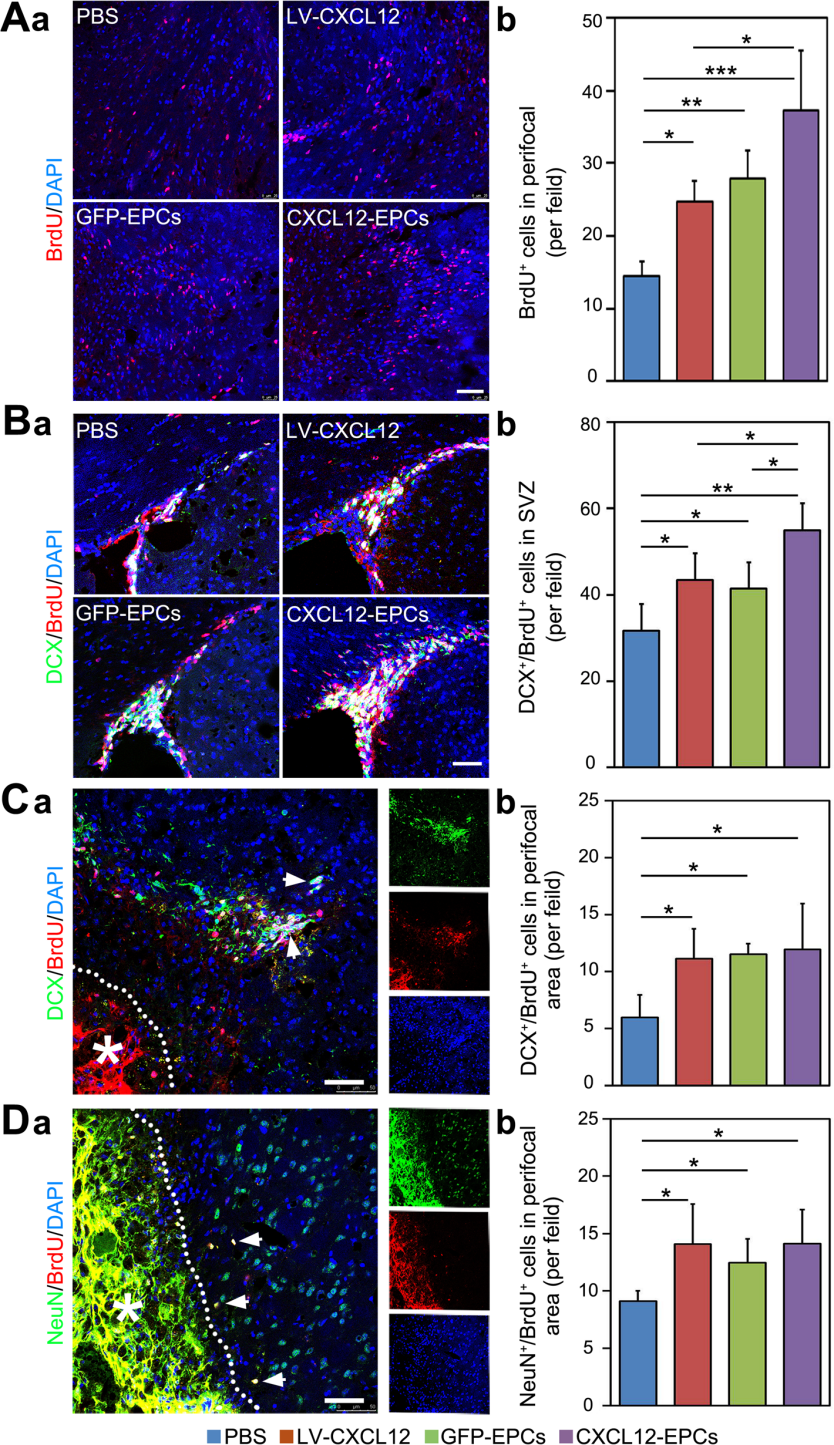
CXCL12-EPC transplantation promoted neurogenesis in ischemic mouse brain. **A** (**a**) BrdU staining and (**b**) quantification of BrdU^+^ cells in perifocal area at 5 weeks after MCAO. **B** (**a**) Double immunostaining of DCX and BrdU-positive cells and (**b**) quantification of DCX^+^/BrdU^+^ cells in the SVZ at 5 weeks after MCAO. **C** (**a**) Double immunostaining of DCX and BrdU cells (white arrows) and (**b**) quantification of DCX^+^/BrdU^+^ cells in perifocal region of ipsilateral hemisphere at 5 weeks after MCAO. **D** (**a**) Double immunostaining of NeuN and BrdU (white arrows indicate double positive cells) and (**b**) quantification of NeuN^+^/BrdU^+^ cells in perifocal region of ipsilateral hemisphere 5 weeks after MCAO. *n* = 3–4 mice/group. Scale bar: 50 μm. Data presented as mean ± SD. *p < 0.05, **p < 0.01. All markers presented by pseudo colors. PBS phosphate-buffered saline, GFP-EPC endothelial progenitor cell modified by *gfp* gene, GFP green fluorescent protein, CXCL12-EPC endothelial progenitor cell modified by *cxcl12* gene, SVZ subventricular zone

The neuroblasts migrate close to blood vessels, and vasculature plays an important role for long-term neurogenesis, so the optimization of vascularization may promote neurogenesis after stroke [39]. BrdU staining were performed to show that the number of newly born cells in treatment groups were significantly increased compared to the PBS control group at 5 weeks after pMCAO, with even higher number of BrdU^+^ cells in the CXCL12-EPC treatment group than the LV-CXCL12 treatment group (Fig. 4A). To analyze whether the transplantation of CXCL12-EPCs promoted focal neurogenesis, which facilitates functional recovery after ischemia [40], double immunostaining of DCX and BrdU was performed. The number of DCX^+^/BrdU^+^ NPCs significantly increased in the SVZ in all therapeutic groups compared to the PBS group, with significantly more DCX^+^/BrdU^+^ cells in the CXCL12-EPC group compared to the LV-CXCL12 and GFP-EPC group (Fig. 4B), which indicated that CXCL12-EPC transplantation promoted the proliferation of NPCs in SVZ. The process of neurogenesis consists of the proliferation, migration, and maturation of NPCs [41, 42]. Further data showed that the number of DCX^+^/BrdU^+^ NPCs (Fig. 4C) and NeuN^+^/BrdU^+^ newborn neurons (Fig. 4D) in the perifocal area of ischemia were significantly increased in all therapeutic groups compared to the PBS group. These results suggest that CXCL12-EPC transplantation augmented neurogenesis, through promoting NPC proliferation at 4 weeks after transplantation.

### CXCL12-EPC transplantation protected myelin sheath integrity in ischemic mouse brain

**Fig. 5:**
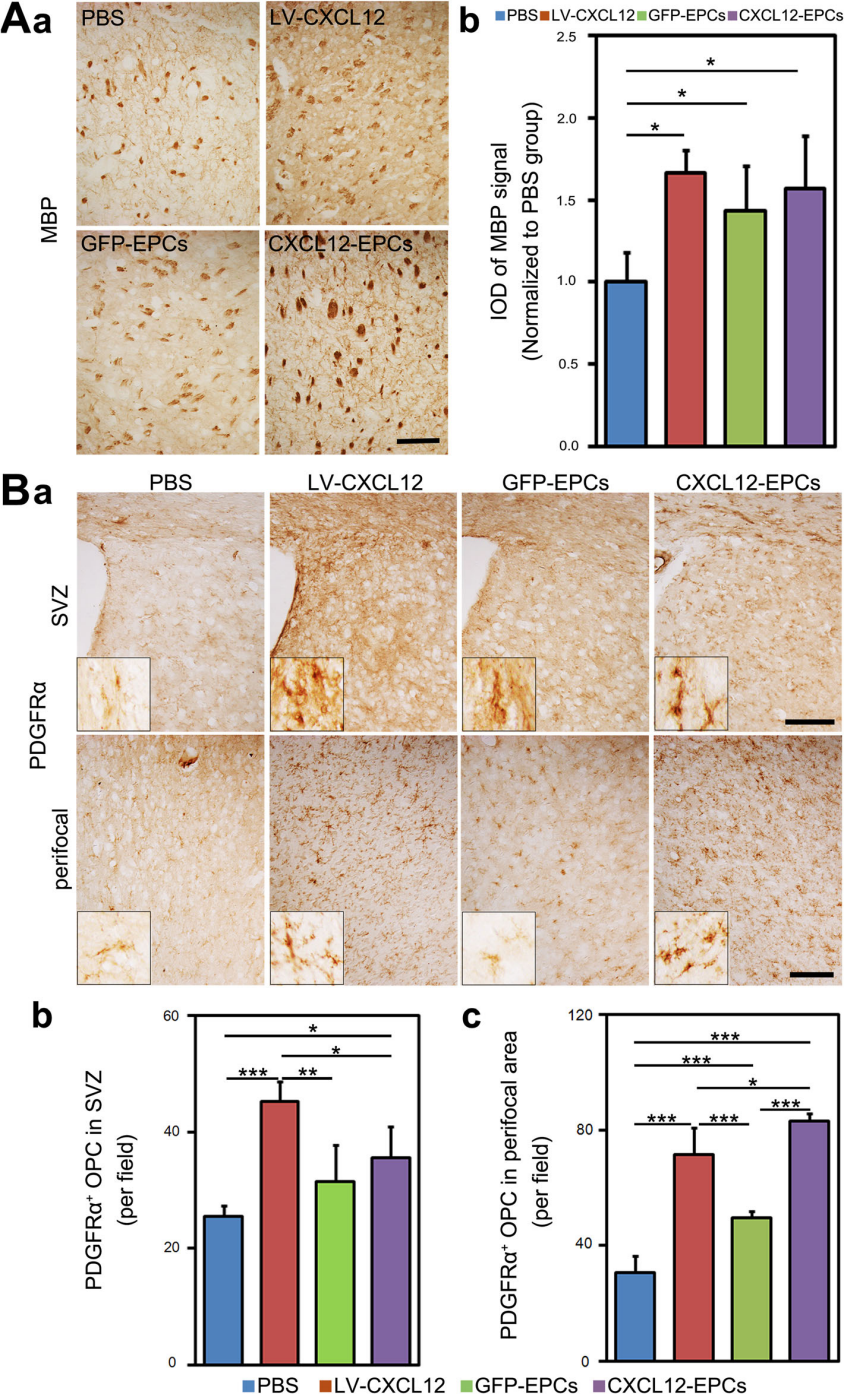
CXCL12-EPC transplantation protected myelin sheath integrity in ischemic mouse brain. **A** (**a**) DAB immunostaining of MBP-positive cells and (**b)** IOD semiquantification of MBP^+^ signal in ipsilateral hemisphere at 5 weeks after MCAO. **B** (**a**) DAB immunostaining of PDGFRα-positive cells in SVZ and perifocal area and quantification of PDGFRα^+^ cells in (**b**) SVZ and (**c**) perifocal area in ipsilateral hemisphere at 5 weeks of ischemic mouse brain. Insets are higher magnifications from corresponding images. *n* = 4 mice/group. Scale bar: 100 μm. Data presented as mean ± SD. *p < 0.05, **p < 0.01, ***p < 0.001. MBP myelin basic protein, PBS phosphate-buffered saline, GFP-EPC endothelial progenitor cell modified by *gfp* gene, GFP green fluorescent protein, CXCL12-EPC endothelial progenitor cell modified by *cxcl12* gene, IOD integral optical density, PDGFR platelet-derived growth factor receptor, OPC oligodendrocyte progenitor cell, SVZ subventricular zone

To demonstrate whether CXCL12-EPC transplantation protects the myelin sheath integrity, which ensures the correct signal transduction and neuronal conductivity [43, 44], MBP immunostaining for myelin sheath protein was performed. As an indicator of myelin sheath integrity, the MBP intensity of the ipsilateral hemisphere in all therapeutic groups was higher than that in the PBS control group (Fig. 5A). Further, we examined whether CXCL12-EPC transplantation influenced the proliferation and migration of OPCs. CXCR4 expression by OPCs has been demonstrated in our previous study, which shows that CXCR4 was expressed abundantly in NG2-positive and/or PDGFRα-positive OPCs. OPCs were separately quantified by staining for PDGFRα^+^ cells in the SVZ and the perifocal area in the ipsilateral hemisphere. The data showed that the number of PDGFRα^+^ cells was significantly increased in all therapeutic groups compared to the PBS control group both in the SVZ and the perifocal area, with more PDGFRα^+^ cells in the SVZ in the LV-CXCL12 group compared to the GFP-EPC and CXCL12-EPC groups and more PDGFRα^+^ cells in the perifocal area in the CXCL12-EPC group than the LV-CXCL12 and GFP-EPC groups (Fig. 5B). These data indicated that CXCL12-EPC transplantation protected brain myelin sheath integrity and promoted the proliferation and migration of OPCs at 4 weeks after transplantation.

### CXCL12-EPCs showed higher proliferation ability, VEGF secretion, and tube formation

**Fig. 6:**
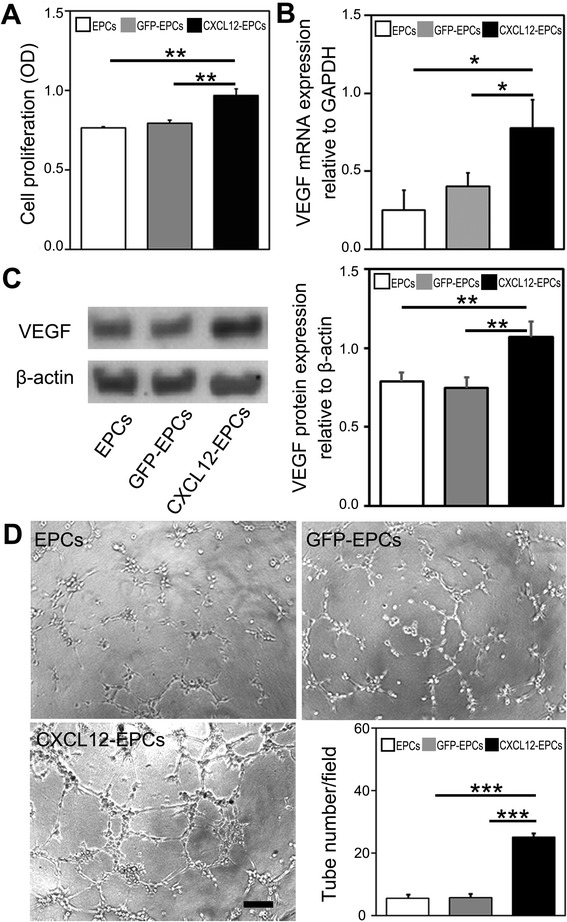
CXCL12-EPCs showed higher proliferation, VEGF secretion, and tube formation ability. **a** CXCL12-EPCs held higher cell proliferation ability. **b** Real-time PCR results show increased expression of VEGF mRNA in CXCL12-EPCs. **c** Western blot results show increased expression of VEGF protein in CXCL12-EPCs and densitometric analysis of VEGF protein expression. **d** Representative images show CXCL12-EPCs had higher tube formation ability. *n* = 3, technical replica. Data presented as mean ± SD. *p < 0.05, **p < 0.01, ***p < 0.001. OD optical density, EPC endothelial progenitor cell, GFP-EPC endothelial progenitor cell modified by *gfp* gene, GFP green fluorescent protein, CXCL12-EPC endothelial progenitor cell modified by *cxcl12* gene, VEGF vascular endothelial growth factor

CXCL12-EPCs exhibited higher cell proliferation ability compared to GFP-EPCs and EPCs based on CCK assay performed using cell culture (Fig. 6a). Previous studies showed that the conditional medium of EPCs contains many kinds of growth factors, including VEGF, basic fibroblast growth factor (bFGF), platelet-derived growth factor (PDGF) [45], and brain-derived neurotrophic factor (BDNF), which contribute to its ability to promote adult SVZ neurogenesis [46]. To investigate whether *cxcl12* modification of EPCs enhanced the secretion of these trophic factors in vitro, real-time PCR was performed to examine the mRNA expression of VEGF, BDNF, bFGF, and PDGF in EPCs, GFP-EPCs, and CXCL12-EPCs. The result showed that CXCL12-EPCs expressed higher levels only of VEGF mRNA compared to EPCs and GFP-EPCs (Fig. 6b, others not shown). Western blot analysis also identified the increased expression of VEGF protein in CXCL12-EPCs (Fig. 6c). We subsequently examined the tube formation ability of these EPCs on Matrigel. The results revealed that CXCL12-EPCs have higher tube formation ability compared to EPCs and GFP-EPCs (Fig. 6d).

## Discussion

Ischemic stroke results in the nonselective damage of all cells in the brain, including endothelial cells, neurons, oligodendrocytes, and so forth [1]. Treatment strategies for ischemic stroke after the subacute phase would have a higher chance of success by promoting angiogenesis, neurogenesis, and remyelination simultaneously. In this study, we demonstrated that the transplantation of *cxcl12* gene-engineered stem cell CXCL12-EPCs into mouse brain at 1 week after ischemia, a considerably delayed treatment regimen, still significantly improved neural behavioral recovery and reduced brain atrophy at 5 weeks after ischemia. The efficacy of the combined treatment of CXCL12-EPCs is significantly higher compared to the single therapies of GFP-EPC stem cell therapy or LV-CXCL12 gene therapy. This strategy significantly extended the treatment window for ischemic stroke.

Mechanistic investigations showed that CXCL12-EPC transplantation greatly increased the blood vessel density and promoted angiogenesis compared to the PBS control group. By using the average number of blood vessels in the ipsilateral hemisphere and the ratio of the number of blood vessels in the ipsilateral to contralateral hemisphere, we demonstrated that both the average number of blood vessels and the ratio were higher in the CXCL12-EPC group compared to the other groups. For the evaluation of angiogenesis, the brain sections were double stained with anti-CD31 (endothelial cell marker) and BrdU (incorporated into newly formed cell nucleus) antibodies to label newly formed blood vessels. The CD31/BrdU double-positive cells were considered new sprouts of blood vessels. Comparing the extent of angiogenesis and the increase of blood vessel density, it is obvious that angiogenesis cannot account for the observed blood vessel density increase. One logical explanation is that CXCL12-EPCs also protected vessels from delayed damage.

In addition to a significant increase of angiogenesis and neurogenesis, the proliferation and migration of OPCs were observed in the CXCL12-EPC-treated group. PDGFRα expression has been found on various types of cells. However, in the nontumor central nervous system, most of the PDGFRα-positive cells were OPCs, then differentiating into oligodendrocytes [47]. Although the highest NPC proliferation was observed with the CXCL12-EPC-treated group, there were no significant differences in the migration and maturation of NPCs in the perifocal region among the three treatment groups. Myelin sheath integrity, as indicated by MBP staining, was also improved by all three treatment regimens without significant differences among the three groups. Studies with longer time course are warranted to further understand the long-term impacts on NPC and OPC migration and maturation by different treatment designs.

In-vitro studies have shown that CXCL12-EPCs presented higher cell proliferation, vascular endothelial growth factor (VEGF) expression, and tube formation ability compared to GFP-EPCs and nonmodified EPCs. In our previous study, it was also shown that CXCL12-EPCs have higher migratory capacity than GFP-EPCs [31]. VEGF protein expression was significantly increased in CXCL12-EPCs compared to EPCs and GFP-EPCs in vitro. It has been reported that the expression of CXCL12 and VEGF as well as the number of EPCs in blood strongly correlated with each other in ischemic patients [48, 49]. Subacute administration of VEGF can promote angiogenesis in the ischemic penumbra and significantly improve neurological recovery in ischemic rats [50]. The combination of CXCL12 and VEGF enhanced angiogenesis more than the monotherapies under conditions of hypercholesterolemia [51], in line with the enhanced effects of CXCL12-EPC cell therapy over LV-CXCL12 gene therapy and GFP-EPC cell therapy alone in protecting blood vessels, promoting the proliferation of NPCs in the SVZ, and the migration of OPCs in the perifocal area of the mouse brain in the weeks after ischemic brain injury. It has been shown both in tumor models [52] and in our previous work in a cell culture model [31] that the CXCL12/CXCR4 axis promotes the expression of VEGF both at the mRNA and protein levels, most likely through the Akt pathway. VEGF not only plays a key role in neuroprotection, angiogenesis, and neurogenesis, but also promotes oligodendrocyte maturation, which may improve the histological and functional outcomes through multiple mechanisms [53–55]. The benefits observed with CXCL12 and CXCL12-EPC treatment could be at least partially attributed to their increased VEGF expression.

One of the challenges of translating stem cell therapy into the clinical setting is the difficulty of tracking the stem cells in vivo at both cellular and molecular levels following an extended time course [56, 57]. In a small-size animal experiment, we tested our hypothesis that *cxcl12*-engineered EPCs will show improved efficacy compared to unmodified EPCs. In the study, very few EPCs were observed at 4 weeks after transplantation, which suggested that the transplanted EPCs probably mainly function through the upregulation of multiple trophic factors, which has been demonstrated in a myocardial injury model [58]. Further study is warranted to follow the transplanted cells at smaller time intervals to gain better insight into their migration, differentiation, and localization fate. Superparamagnetic iron oxide nanoparticles as well as I^125^ isotopes have been successfully used in tracking stem cells in vivo [13, 59]. Further studies using such tracking means will better inform on the fate and potential mechanisms of function of the transplanted cells.

## Conclusion

This study showed that *cxcl12* engineering of EPCs resulted in better histological and functional outcomes in ischemic mice, even when they are transplanted at 1 week after pMCAO. These beneficial effects may be related to the protection of blood vessels and myelin sheath integrity, the promotion of angiogenesis and neurogenesis, and the enhancement of the proliferation and migration of OPCs during the 4 weeks after treatment. These data suggest that *cxcl12* modification of EPCs is a promising strategy in the treatment of ischemic stroke with a wider treatment window during the post-acute phase after ischemic injury.

## Abbreviations

BDNF: Brain-derived neurotrophic factor
bFGF: Basic fibroblast growth factor
EPC: Endothelial progenitor cell
GFP: Green fluorescent protein
IOD: Integral optical density
MBP: Myelin basic protein
mNSS: Modified neurological severity score
NPC: Neural progenitor cell
OPC: Oligodendrocyte progenitor cell
PBS: Phosphate-buffered saline
PDGF: Platelet-derived growth factor
pMCAO: Permanent middle cerebral artery occlusion
SVZ: Subventricular zone
VEGF: Vascular endothelial growth factor
